# CDC Deployments to State, Tribal, Local, and Territorial Health Departments for COVID-19 Emergency Public Health Response — United States, January 21–July 25, 2020

**DOI:** 10.15585/mmwr.mm6939a3

**Published:** 2020-10-02

**Authors:** Emilio Dirlikov, Ethan Fechter-Leggett, Stacy L. Thorne, Caitlin M. Worrell, Jennifer C. Smith-Grant, Jonathan Chang, Alexandra M. Oster, Adam Bjork, Stanley Young, Alvina U. Perez, Tricia Aden, Mark Anderson, Susan Farrall, Jaime Jones-Wormley, Katherine Hendricks Walters, Tanya T. LeBlanc, Rebecca Greco Kone, David Hunter, Laura A. Cooley, Vikram Krishnasamy, Jennifer Fuld, Carolina Luna-Pinto, Tanya Williams, Ann O’Connor, Randall J. Nett, Julie Villanueva, Nadia L. Oussayef, Henry T. Walke, Jill M. Shugart, Margaret A. Honein, Dale A. Rose, CDC COVID-19 State, Tribal, Local, and Territorial Response Team, CDC COVID-19 State, Tribal, Local, and Territorial Response Team, Noelle Anderson, CDC; David Bang, Terrika Barham, Shaliondel Benton, Amy Blain, Mary Boyd, Bruce Bradley, Shakia Bright, Michael Bruce, Victor Cabada, Georgina Castro, Dena Cherry-Brown, Erik Coleman, Janet Cowins, Pamela Craig, Johnni Daniel, Darlene Davis, Stacy De, Naomi Drexler, Jessica Dull, Sherry Farr, Phillip Finley, Karrie Finn, Denise Freeman, Corinne Fukayama, Nicole Gaarenstroom, Micha Ghertner, Maleeka Glover, Gail Grant, Sean Griffing, DeMoncheri Harris, Diane Harris, Nikki Hayes, Seung Hee, Corey Henry, Donna Henry, Janine Hines, Amy Hudson, Kashif Iqbal, Jennifer Isenberg, Mary Jenkins, Charlotte Kabore, Sandor Karpathy, Daphne Kennebrew, Karen Kun, Ryan Lash, Rene Lavinghouze, Rachel Leavitt, Sooji Lee, Eva Leidman, Oscar Leon, Sarah Leonard, Garry Lowry, Elizabeth Lundeen, Mechele Lynch, Michon Mabry, Jana Manning, Kelsey McCall, Henraya McGruder, Sarah Merkle, Jenna Meyer, Patrick Moonan, Jazmyn Moore, Pamelian Norwood, Seseni Nu, John Oeltmann, Krishna Palipudi, Monica Parise, Ritchard Parry, Abrienne Patta, Chandra Pendergraft, Kristen Pettrone, Heidi Pfeifer, Tracy Powell, Nykiconia Preacely, Yanping Qi, Jessica Ricaldi, Regina Richardson-Moore, LaShonda Roberson, Sergio Rodriguez, Tomas Rodriguez, Andrew Ruiz, Sharon Saydah, Abdoulie Senesie, Connie Sexton, Shari Shanklin, Christopher Sieradzki, Amberia Simpson, De’Lisa Simpson, Stephanie Snodgrass, Lisa Speissegger, Alisa Spieckerman, Danielle Stollar, Nimalie Stone, Brittany Sunshine, Philana Swann, Rezwana Uddin, Diana Valencia, Chastity Walker, Malaika Washington, Seh Welch, Shawna Williams, Rebecca Woodruff, Evonne Woodson, Graydon Yatabe, Hussain Yusuf

**Affiliations:** ^1^CDC COVID-19 Emergency Response Team; ^2^Division of Emergency Operations, CDC.; CDC; CDC; CDC; CDC; CDC; CDC; CDC; CDC; CDC; CDC; CDC; CDC; CDC; CDC; CDC; CDC; CDC; CDC; CDC; CDC; CDC; CDC; CDC; CDC; CDC; CDC; CDC; CDC; CDC; CDC; CDC; CDC; CDC; CDC; CDC; CDC; CDC; CDC; CDC; CDC; CDC; CDC; CDC; CDC; CDC; CDC; CDC; CDC; CDC; CDC; CDC; CDC; CDC; CDC; CDC; CDC; CDC; CDC; CDC; CDC; CDC; CDC; CDC; CDC; CDC; CDC; CDC; CDC; CDC; CDC; CDC; CDC; CDC; CDC; CDC; CDC; CDC; CDC; CDC; CDC; CDC; CDC; CDC; CDC; CDC; CDC; CDC; CDC; CDC; CDC; CDC; CDC; CDC; CDC; CDC; CDC; CDC; CDC; CDC; CDC; CDC; CDC; CDC; CDC; CDC.

Coronavirus disease 2019 (COVID-19) is a viral respiratory illness caused by SARS-CoV-2. During January 21–July 25, 2020, in response to official requests for assistance with COVID-19 emergency public health response activities, CDC deployed 208 teams to assist 55 state, tribal, local, and territorial health departments. CDC deployment data were analyzed to summarize activities by deployed CDC teams in assisting state, tribal, local, and territorial health departments to identify and implement measures to contain SARS-CoV-2 transmission (*1*). Deployed teams assisted with the investigation of transmission in high-risk congregate settings, such as long-term care facilities (53 deployments; 26% of total), food processing facilities (24; 12%), correctional facilities (12; 6%), and settings that provide services to persons experiencing homelessness (10; 5%). Among the 208 deployed teams, 178 (85%) provided assistance to state health departments, 12 (6%) to tribal health departments, 10 (5%) to local health departments, and eight (4%) to territorial health departments. CDC collaborations with health departments have strengthened local capacity and provided outbreak response support. Collaborations focused attention on health equity issues among disproportionately affected populations (e.g., racial and ethnic minority populations, essential frontline workers, and persons experiencing homelessness) and through a place-based focus (e.g., persons living in rural or frontier areas). These collaborations also facilitated enhanced characterization of COVID-19 epidemiology, directly contributing to CDC data-informed guidance, including guidance for serial testing as a containment strategy in high-risk congregate settings, targeted interventions and prevention efforts among workers at food processing facilities, and social distancing.

## CDC Deployments to Assist Health Departments

On January 21, 2020, CDC activated its Emergency Operations Center to facilitate coordination for domestic and international COVID-19 response efforts (*2*); the same day, at the request of the Washington State Health Department, CDC deployed a team to Washington to support the health department’s epidemiologic investigation of the first U.S. case of COVID-19 in a traveler returning from China (*3*). On March 15, CDC established a dedicated COVID-19 response section to support state, tribal, local, and territorial health departments (*4*). CDC deployment data were analyzed to describe activities by deployed CDC teams in assisting state, tribal, local, and territorial health departments in the identification and implementation of measures to contain SARS-CoV-2 transmission (*1*). The CDC COVID-19 state, tribal, local, and territorial response section provides support to health departments by responding to inquiries, identifying and collaborating with CDC subject matter experts, and deploying CDC teams in response to receipt of official requests for assistance from health departments. Dedicated teams of CDC subject matter experts have participated in evaluating contact tracing efforts and have investigated COVID-19 epidemiology in counties with rapidly increasing numbers of cases and incidence (“hotspots”) to identify jurisdictions needing targeted support (*5*). Further, the CDC COVID-19 state, tribal, local, and territorial response section helps coordinate efforts between CDC, health departments, and subject matter experts across federal agencies and other organizations including the CDC Foundation, the National Association of County and City Health Officials, the Association of Public Health Laboratories, Association of State and Territorial Health Officials, and the Council of State and Territorial Epidemiologists.

The CDC COVID-19 state, tribal, local, and territorial response section coordinated deployment requirements with health departments and selected staff members with the necessary skills after an official request for assistance. CDC COVID-19 Response General Staff, Division of Emergency Operations, and Office of Safety, Security, and Asset Management ensured that all deployers were supported before, during, and after deployment, including providing briefings before and after deployments; coordinating risk assessments, medical clearance, and travel and lodging arrangements; and issuing deployment-essential equipment, including personal protective equipment to prevent SARS-CoV-2 transmission during field deployments. Deployer feedback received during postdeployment debriefings were used to improve deployment processes and procedures for subsequent deployments.

**FIGURE 1:**
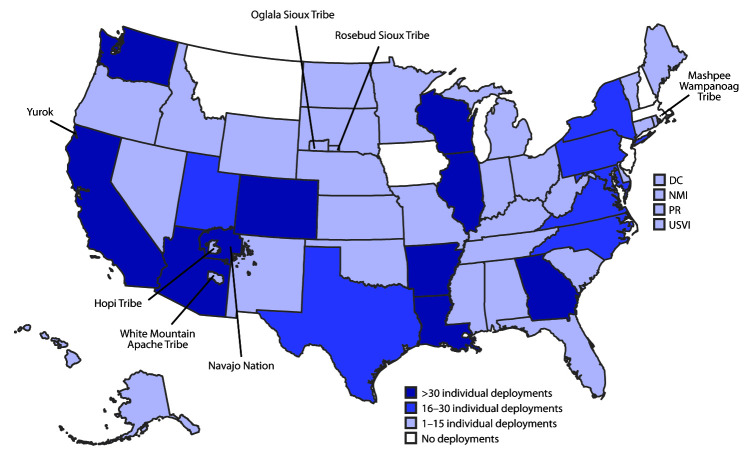
Location of deployments* by CDC staff members to state, tribal, local, and territorial health departments — United States, January 21–July 25, 2020 **Abbreviations:** DC = District of Columbia; NMI = Northern Mariana Islands; PR = Puerto Rico; USVI = U.S. Virgin Islands. * 726 CDC staff members deployed on 208 teams, as part of 1,009 total deployments (individual staff members could deploy more than one time).

**FIGURE 2:**
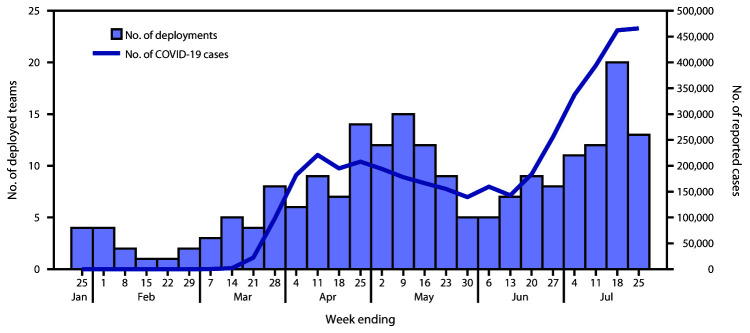
Number of CDC deployment teams to state, tribal, local, and territorial health departments and reported COVID-19 cases, by week — United States, week 4–30 (N = 208 teams)* **Abbreviation**: COVID-19 = coronavirus disease 2019. * Does not include deployments to U.S. quarantine stations and airports, repatriations centers, as part of outbreak response on cruise ships, or other response teams.

During January 21–July 25, in response to official requests for assistance, 1,009 CDC staff members participated in 208 CDC deployment teams to assist 55 state, tribal, local, and territorial health departments with COVID-19 emergency public health response activities (Figure 1)*; some persons deployed multiple times. Trends in the deployment of CDC teams generally followed trends in national COVID-19 case counts. The number of deployed field teams per week increased during January–April and declined during May–June; however, from mid-June to July 25, the number of deployed teams increased (Figure 2).

Among 168 (81%) teams that had completed deployment by July 25, the mean deployment duration was 20 days (range 1–89 days) (Table). Among the remaining 40 teams deployed as of July 25, duration of deployment ranged from 1–146 days; several teams were providing sustained epidemiologic support. Among the 208 teams deployed following official requests for assistance, 178 (85%) provided assistance to state health departments, 12 (6%) to tribal health departments, 10 (5%) to local health departments, and eight (4%) to territorial health departments.

**TABLE:** Summary of CDC deployment teams* and staff members to state, tribal, local, and territorial health departments for COVID-19 emergency public health response — United States, January 21–July 25, 2020

Characteristic	No. (%)
**Total teams**	208 (100)
**Team deployment duration, mean days (range)**	
Completed deployment (168 teams)	20 (1–89)
Currently deployed as of July 25 (40 teams)	48 (1–146)
**Teams by jurisdiction type**	
State	178 (85)
Tribal	12 (6)
Local	10 (5)
Territorial	8 (4)
**Teams by types of technical assistance provided^†^**	
Epidemiology	144 (69)
Infection prevention and control in health care settings	77 (37)
Health communications	37 (18)
Community mitigation	36 (17)
Occupational safety and health	31 (15)
Laboratory	21 (10)
Surge support	9 (4)
Information technology	8 (4)
**Teams that assisted with investigating transmission in high-risk congregate settings**	
Total^§^	87 (42)
Long-term care facilities	53 (26)
Food processing facilities	24 (12)
Correctional facilities	12 (6)
Settings that provide services to persons experiencing homelessness	10 (5)
Deployed staff members*	
Total individual deployments	1,009
Total individual CDC staff members who deployed	726
**No. of times individual staff members deployed^¶^**	
1	516 (71)
2	156 (21)
3	40 (6)
4	9 (1)
5	5 (1)
**Primary deployer role among total individual deployments**	
Epidemiologic support	422 (42)
Leadership**	137 (14)
Infection prevention and control	88 (9)
Clinical support^††^	65 (6)
Data science	59 (6)
Laboratory science	47 (5)
Health communications and community outreach	46 (5)
Subject matter expertise^§§^	36 (4)
Occupational safety and health	31 (3)
Coordination	28 (3)
Veterinary science	11 (1)
Behavioral science	12 (1)
Other^¶¶^	27 (3)

**Abbreviation:** COVID-19 = coronavirus disease 2019.

* Deployments through CDC COVID-19 health department response section. Does not include deployments to U.S. quarantine stations and airports, repatriations centers, as part of outbreak response on cruise ships, or other response teams. Some individual CDC staff members were deployed more than once.

^†^ Deployed teams provided a diversity of technical assistance, and a single team could assist with more than one area of technical assistance.

^§^ Total differs from sum of all high-risk congregate settings because some teams worked in multiple high-risk congregate settings.

^¶^ Percent represents percentage of total CDC staff members who deployed.

** Leadership includes staff members with any deployment roles listed as “Senior,” “Lead,” “Deputy,” “Team Lead,” “Co-Lead,” or “Deputy Lead,” with leadership staff member classification superseding all other classifications.

^††^ Clinical support includes staff members who were physicians, nurses, or pharmacists who were not listed with an alternate primary deployer role.

^§§^ Subject matter expertise includes staff members with any deployment roles listed as “SME,” “Specialist,” and deployments under COVID-19 Resource Assistance Field Team and Centers for Medicare & Medicaid Services teams.

^¶¶^ Other includes staff members listed as vessel sanitation, technical assistance, focus groups, and individual deployments that could not otherwise be classified (n = 23).

Because state, tribal, local, and territorial health departments could request assistance with a range of public health activities, deployed team members possessed diverse technical skills and expertise, and a single team could provide technical assistance in multiple areas. The top five areas of technical assistance provided by deployed teams were the following: 1) epidemiologic support (144 teams; 69%), 2) infection prevention and control in health care settings (77; 37%), 3) health communications (37; 18%), 4) community mitigation (36; 17%), and 5) occupational safety and health (31; 15%). Some deployed CDC teams provided subject matter expertise in investigation and mitigation of SARS-CoV-2 transmission in high-risk congregate settings, which often include populations at increased risk for severe COVID-19–associated outcomes, such as long-term care facilities (53 teams; 26%), food processing facilities (24 teams; 12%), correctional facilities (12; 6%), and settings that provide services to persons experiencing homelessness (10; 5%). Knowledge, attitudes, and practices surveys helped involve community members in identifying barriers to services, difficulties experienced when trying to follow prevention actions, and preferred communication channels. Aligned with CDC’s COVID-19 health equity strategy,^†^ some teams focused attention on supporting local officials in describing health equity issues, such as describing SARS-CoV-2 transmission among disproportionately affected racial and ethnic minority populations, essential frontline workers, persons experiencing homelessness, as well as through a place-based focus, such as responding to COVID-19 outbreaks in rural communities and frontier areas. Twenty-eight (13%) teams deployed specifically to assist in addressing SARS-CoV-2 transmission among racial and ethnic minority groups, including supporting tribal health departments and those focused on COVID-19 among migrant farm workers.

Because CDC staff members could deploy more than once, the 1,009 CDC staff member deployments included 726 individual CDC staff members. Overall, 516 (71%) staff members deployed once, 156 (21%) deployed twice, and 54 (8%) deployed three or more times. Among the 1,009 individual deployments, the top four primary deployer roles were epidemiologic support (422; 42%), leadership (137; 14%), infection prevention and control (88; 9%), and clinical support (65; 6%); additional primary deployer roles included data science, laboratory science, health communications and community outreach, occupational safety and health, coordination, veterinary science, and behavioral science. Deployed CDC staff members helped increase local capacity by assisting with developing data collection instruments, conducting trainings on COVID-19 case investigation and contact tracing, and providing support to improve public health information technology systems.

## Discussion

CDC continues to respond to official requests for assistance from state, tribal, local, and territorial health departments toward supporting COVID-19 emergency public health response activities, including through the deployment of CDC staff members. CDC deployments were responsive to evolving public health needs, as reflected by similar trends in number of deployed teams and reported national case counts. Approximately 700 CDC staff members deployed, and approximately one half of individual deployments were completed by staff members who had deployed more than once. On average, teams deployed for nearly 3 weeks, and several teams provided more sustained support.

Collaborations between health departments and CDC have provided critical information for developing new or revised national guidance including improved mitigation strategies (https://www.cdc.gov/coronavirus/2019-ncov/communication/guidance-list.html). For example, CDC and health departments developed and implemented the use of serial testing as a successful containment strategy, which was used to interrupt transmission in long-term care facilities in Washington^§^ (*6*), in correctional and detention facilities in Louisiana^¶^ (*7*), and among residents and staff members of homeless shelters in Washington** (*8*). Multijurisdictional support helped describe the need for targeted interventions and prevention efforts among workers at food processing facilities, including an analysis of COVID-19 cases among meat and poultry processing facility workers in 23 states that found that among cases with race/ethnicity reported, 87% occurred among racial or ethnic minorities^††^ (*9*). More generally, deployed teams assisted health departments conduct epidemiologic investigation after outbreaks associated with social gatherings, such as cases and deaths resulting from transmission at two family gatherings in Chicago (*10*); the results of these investigations helped support and refine CDC COVID-19 recommendations on social distancing. The impact of collaborations extends beyond health agencies. For example, on April 2, the Centers for Medicare & Medicaid Services and CDC issued guidance to implement universal testing of long-term care facility residents, covered through Medicare, as an effective containment strategy, based on collaborative work between CDC and health departments, including in King County, Washington^§§^ (*6*). On July 30, Tyson Foods, the world’s second largest processor of chicken, beef, and pork, announced it would expand weekly COVID-19 testing and symptom monitoring among employees as part of a nationwide strategy to contain infections, per CDC guidance^¶¶^ and after data analysis conducted in collaboration with 23 state health departments (*9*). Among 90 total COVID-19–related reports published in *MMWR* up to the August 28th issue, 30 (33%) resulted from these deployments.

The findings in this report are subject to at least two limitations. First, deployment data could be subject to data quality issues, despite regular data reviews and a full review of individual deployment data for this report. Second, this report describes deployments through the CDC COVID-19 state, tribal, local, and territorial response section. Health departments were also supported by other CDC COVID-19 response sections, as well as by CDC staff members already working within state, tribal, local, and territorial departments of health, such as Career Epidemiology Field Officers, Public Health Associates,*** and Epidemic Intelligence Service Officers.^†††^ In addition, during January 17–July 25, 2020, CDC deployed 513 staff members to U.S. quarantine stations and airports as well as 159 staff members to support repatriation missions.^§§§^

As the COVID-19 pandemic continues, ongoing collaboration between health departments and CDC will aim to strengthen local capacity, assist with outbreak response, and, as new evidence emerges, directly contribute to data-informed guidance that will benefit local and national response efforts.

**Summary**

**What is already known about this topic?**

As part of the COVID-19 emergency public health response, CDC deploys field teams upon request to assist state, tribal, local, and territorial health departments.

**What is added by this report?**

As of July 25, 2020, CDC had deployed 208 teams to assist 55 state, tribal, local, and territorial health departments. Teams worked with local counterparts to address transmission in high-risk settings, including long-term care facilities (26%), food processing facilities (12%), correctional facilities (6%), and settings providing services to persons experiencing homelessness (5%).

**What are the implications for public health practice?**

CDC collaborations with health departments have strengthened local capacity, assisted with outbreak response, and directly contributed to data-informed guidance, benefiting local and national response efforts.

## Footnotes

* Includes both in-field and remote deployments and does not include CDC staff members deployed to U.S. quarantine stations and airports, repatriations centers, or as part of outbreak response on cruise ships.

† https://www.cdc.gov/coronavirus/2019-ncov/downloads/community/CDC-Strategy.pdf.

§ https://www.cdc.gov/longtermcare/index.html.

¶ https://www.cdc.gov/coronavirus/2019-ncov/community/correction-detention/guidance-correctional-detention.html.

** https://www.cdc.gov/coronavirus/2019-ncov/community/homeless-shelters/plan-prepare-respond.html.

†† https://www.cdc.gov/coronavirus/2019-ncov/community/organizations/meat-poultry-processing-workers-employers.html.

§§ https://www.cms.gov/files/document/4220-covid-19-long-term-care-facility-guidance.pdf.

¶¶ https://www.meatpoultry.com/articles/23549-tyson-expanding-covid-19-testing-monitoring-as-part-of-nationwide-strategy.

*** https://www.cdc.gov/phap/index.html.

††† https://www.cdc.gov/eis/index.html.

§§§ https://www.cdc.gov/coronavirus/2019-ncov/php/open-america/staffing.html.
